# Fruit and vegetable preservation from classical to advanced nanotechnology: an overview of efficacy and health concerns

**DOI:** 10.1016/j.fochx.2025.102984

**Published:** 2025-09-03

**Authors:** Mohammed I. Alquraishi, Nora A. Alfadda, Wajude A. Alabdullatif, Chinnadurai Veeramani, Ahmed S. El Newehy, Khalid S. Al-Numair, Amal A. Aloud, Mohammed A. Alsaif

**Affiliations:** a Department of Community Health Sciences, College of Applied Medical Sciences, King Saud University, P.O. Box 10219, Riyadh 11433, Saudi Arabia; b Department of Food Sciences and Nutrition, College of Food and Agriculture Sciences, King Saud University, Riyadh, Saudi Arabia

**Keywords:** Fruits, Vegetables, Nanoparticles, Edible coating, Chitosan, Zinc oxide, Silver, Calcium nanoparticles

## Abstract

For a long time, classical food preservation methods have been employed to control deterioration in fruits and vegetables. However, these methods exhibit several limitations and may not be entirely successful in managing food waste. Lately, nanotechnology has emerged as a novel approach to compensate for the disadvantages of currently used methods. Eventually, nanotechnology utilization is still in its infancy and requires rigorous evaluation. In this review, we aimed to explore the efficacy of nanotechnology in preserving fresh produce and highlight its potential applications. Apparently, various nanoparticles applied during pre or postharvest significantly extended the shelf life of fruits and vegetables. Choosing the appropriate nanoparticles and mode of application might significantly impact their effectiveness. Yet, concerns regarding nanoparticles' safety and commercial availability could hinder their broad applications. In summary, nanoparticles alone or combined with classical methods hold promise in preserving fresh produce, but more efforts are required to advance their practical usage.

## Highlights

• Classical approaches to food preservation require optimization to reduce fresh produce loss.
• Nanotechnology holds promise as an alternative strategy to extend fresh produce shelf life.
• Conjugated usage of pre and postharvest nanoparticles serves as a new venue for optimal preservation.
• Future studies should focus on the safety of newly synthesized nanoparticles.
• Future studies are encouraged to utilize biological models to ensure translation applicability.

## 1 Introduction

The global human population has grown significantly over the past few decades. Although the population growth rate varies over the years, the increment of population growth is projected to continue and reach approximately 9.7 billion by 2050 (Gu et al., 2021). Ultimately, population growth is parallel with increased global food demand. A recent prediction report estimated the increase in food demand to reach 56 % by 2050, highlighting the potential risks to food security and global hunger (van Dijk et al., 2021). Moreover, the unexpected temperature rise has an adverse effect on major crop yields. In various countries, decreased crop yields due to climate change have already been observed (Feng et al., 2023). In fact, altered environmental factors such as elevated heat and CO2 will be a substantial challenge that hinders crop yield production from meeting the surge in global food demands (Rezaei et al., 2023). Thus, minimizing food waste, especially postharvest loss, might be critical for food security. Indeed, governments have already developed policies and initiative measures to manage food waste (Santeramo, 2021). Subsequently, enhanced preservation methods might be crucial to minimize food waste, especially in developing countries (Wani et al., 2024).

Conventional methods of food preservation are intended to manage physiological, chemical, and microbiological elements that lead to the decline in food quality. The primary goal of these methods is to reduce spoiling and microbial invasion while extending fresh produce shelf life. In fact, these methods are critical for maintaining flavor and texture, retaining nutritional value, and, most importantly, minimizing food waste (Mafe et al., 2024). These methods include protective coatings (Blancas-Benitez et al., 2022; Perez-Vazquez et al., 2023), minimal food processing (De Corato, 2020), cold storage (Yenare et al., 2024), controlled atmosphere storage (CAS) (Fang & Wakisaka, 2021), canning, dehydration (Mafe et al., 2024; Sridhar et al., 2021), and pickling (Behera et al., 2020). Compared to other food categories, fruits and vegetables exhibit the highest rate of food loss (Cassani & Gomez-Zavaglia, 2022). This increase in loss rate may occur because they fall into the perishable category, which requires extra precautionary measures (Ghoshal, 2018). In addition, fresh produce is usually processed, which potentiates the likelihood of spoilage and eventually leads to diminished food quality parameters (Yousuf et al., 2020). Moreover, processed products are more exposed to environmental factors and become appealing to foodborne microbes, thereby posing a substantial public health concern (Łepecka et al., 2022; Yousuf et al., 2020). To that end, classical preservation methods must be advanced to optimize fruits and vegetables' shelf life and satisfy consumers.

Recently, researchers have been motivated to find alternative solutions to overcome issues related to conventional preservation methods. One of these solutions was nanotechnology, which refers to using particles ranging in size from around 1 to 100 nm (Joudeh & Linke, 2022; Szczyglewska et al., 2023). Nanotechnology has been proposed and applied as an innovative alternative solution. Apparently, nanotechnology alone or in combination with other preservation methods may aid in improving food quality parameters while extending storage duration and reducing cost (Biswas et al., 2022; Shan et al., 2023). Yet, fresh fruits and vegetables could benefit from nanotechnology by advancing bio-coating techniques, which provide a healthier and more sustainable method compared to the classical preservation approaches (Odetayo et al., 2022; Ungureanu et al., 2023). Moreover, bio-coating has a positive environmental impact since it could replace plastic-based packaging materials (Samir et al., 2022). Lately, numerous studies have evaluated edible films and coatings containing nanoparticles (NPs) in preserving the shelf life of fruits and vegetables (Alli et al., 2023; Dulta, Koşarsoy Ağçeli, Thakur, et al., 2022; Kumari et al., 2021; Liu et al., 2020; Rasheed et al., 2023). Yet, nanotechnology usage is still in its infancy and requires rigorous evaluation and further exploration, with a focus on the efficacy and safety of nanoparticles.

Unlike other food items, perishable foods, including fruits and vegetables, are more prone to spoilage. Therefore, evaluating existing and innovative preservation methods is necessary to better understand the impact of these techniques on the quality, sensory attributes, and consumer acceptability of fresh produce. Additionally, evaluating the current literature may aid in shaping future research directions of nanotechnology in extending the shelf life of fruits and vegetables. For this reason, our goal is to provide the scientific community with the most up-to-date knowledge regarding existing preservation techniques and thoroughly discuss recent advancements and the emerging roles of nanotechnology, focusing on potential benefits and existing limitations.

## 2 Classical approaches to food preservation

Harmful microorganisms can lead to the deterioration and loss of quality in fruits and vegetables (Wang & Teplitski, 2023). Therefore, minimizing microorganisms' growth would meet consumers' preferences for food items with a longer shelf life and high nutritional value. Classically, fresh produce can be preserved using various thermal and non-thermal techniques, such as drying, heat treatment, ohmic and high-pressure processing, among others (Boateng, 2023). Each of these techniques has its own strengths and weaknesses. For instance, non-thermal methods have less impact on the overall sensory or nutritional content of the product compared to thermal techniques (Kohli & Shahi, 2017). Thus, using chemicals such as calcium chloride (CaCl2) (Mu et al., 2022) and hydrogen sulfide (HS) combined with thermal methods might yield better preservation outcomes (Ali et al., 2019). This section will briefly discuss the most common existing fruit and vegetable preservation methods and highlight their benefits, limitations, and potential advancements.

Cold storage and freezing have been used for a long time to minimize foodborne illness-causing bacteria and rotting and are considered to be one of the gold standard methods for preserving food (Alegbeleye et al., 2022; Grover & Negi, 2023). In this approach, temperature is usually lowered to inhibit endogenous enzymatic activity, thereby extending the storage duration of fruit and vegetables. Although this method is efficient in preserving the quality of produce for a long time, the temperature needs to be adjusted for each item to avoid chilling injury. In fact, the formation of ice crystals can cause permanent physiological damage to the preserved items despite the recent efforts conducted to optimize the freezing-thawing cycles (Grover & Negi, 2023). Moreover, lowering temperature could be combined with other preserving techniques such as protective coating and CAS to ensure optimized preservation. The CAS method is centralized around regulating the storage gas composition, such as O2 and CO2, thereby reducing the fresh produce metabolic activity and extending their storage life (Makule et al., 2022). Although this approach is practical in certain situations, it may still cause low O2 and CO2 injury (Arshad et al., 2023). In addition, CAS could be expensive and may require sophisticated technology to regulate gas supply.

Food has been traditionally preserved by blanching at elevated temperatures and sealing in airtight jars in a process known as canning. The high temperature creates a sterile environment that inhibits recontamination by killing germs and deactivating rotting enzymes. Fruits and vegetables can be canned and kept for months or even years at room temperature, making it a common method of food preservation. Moreover, food can undergo beneficial alterations by canning, including developing new flavors, deactivating anti-nutritional components, and softening hard tissue (Lee, 2018). Nevertheless, these modifications could also produce unfavorable outcomes, like nutritional loss (Soni & Brightwell, 2022) and changes in color, texture, or flavor. In addition, unwanted chemical reactions can result in food coloring, pectin breakdown, and rancidity of lipids. Thus, oxidation may result during canning and lead to the loss of vitamins and ascorbic acid (Lee, 2018). Moreover, thermal processing may induce the formation of advanced glycation end-products (AGEs), which have a detrimental effect on both nutritional value and human health (Zhang, Li, et al., 2024). Similarly, before being heated, fruits and vegetables are soaked in salt, acetic acid (vinegar), and some spices to avoid microbial growth. Pickled goods are kept fresh by boiling, salting, and/or acidifying them. Applying heat along with vinegar would inhibit the growth of microbiological pathogens. Interestingly, pickled goods may confer multiple health and nutritional benefits. However, pathogenic bacterial strains of *E. coli* and *Salmonella spp.* are resistant to acidic pH, which raises questions regarding the safety of pickled foods with an acidic pH (Behera et al., 2020).

Although conventional preservation methods heavily depend on thermal techniques, it is worth noting that they still exhibit various drawbacks that can affect the nutritional value and quality of fresh produce (Gao, Xia, et al., 2022). Increasing temperatures is a common practice to inhibit microbial growth, but it also leads to a reduction in bioactive components (Wu et al., 2024). In fact, elevated heat for processing purposes may exceed the heat capacity of these components, potentially leading to their destruction, especially in heat-sensitive antioxidants. However, under certain conditions, heating is suggested to break down plant cell compartments and release antioxidants (Toydemir et al., 2022). In addition, heating may cause substantial changes in flavonoid stability and structure (Lin & Xiao, 2024). Nevertheless, lowering the temperature is well known to maintain antioxidants, but extended exposure could cause chilling injury, which may have an adverse effect. Furthermore, chilling injury is associated with altering flavors due in part to changes in indigenous metabolism (Yuan et al., 2024) or altered gene expression and DNA methylation (Duan et al., 2023). Changes in sensory attributes caused by chilling injury are evident in various fruits, including peaches (Farcuh & Hopfer, 2023; Wang et al., 2025), and strawberries (Liu, Liu, et al., 2025), among others. Thus, using non-thermal methods in conjunction with thermal techniques might resolve these issues (Chaudhary et al., 2025). However, non-thermal methods still require sophisticated techniques, skilled personnel, and could be expensive (Asl et al., 2022), concomitant with inducing undesirable changes such as lipid oxidation under certain conditions (Barbhuiya et al., 2021).

Protective coating is another approach that could successfully extend the shelf life of fruits and vegetables. Chitosan is among the most utilized substances to form edible coatings and is considered safe for consumers and the environment. Indeed, chitosan is classified as a safe substance by the US Food and Drug Administration (USFDA) (Kumarihami et al., 2022). Chitosan inhibits the growth of pathogens and could be used to prevent or treat postharvest illnesses by constructing a protective layer on the product surface to eliminate the effect of environmental factors (Adiletta et al., 2021). Moreover, this layer could also serve as a base to incorporate functional foods such as vitamins and minerals (Duan et al., 2019). However, the mechanical characteristics of the chitosan coating significantly limit its commercial usage. Since chitosan is a natural material, it is challenging to undergo extrusion or molding processes and could degrade before reaching its melting point. Also, chitosan is mainly indicated as hydrophilic, the product water activity and the relative humidity of the surrounding air can have an adverse effect on chitosan coatings (Cazón & Vázquez, 2020).

In addition, reducing the moisture level of fruits and vegetables could be another approach to extend their shelf life. This approach has been used since the ancient era and has received substantial development over time. Dehydration is a good example of this approach, where fresh produce is exposed to the sun, hot air, or other drying agents and devices until the moisture content is reduced to prevent enzymatic activity and microbial growth (Chibuzo et al., 2021). Subsequently, dried food can be transported and stored in a cost-effective manner, but the food's nutritional and sensory qualities are significantly diminished. However, fresh produce loses its juicy freshness and becomes rough and fibrous in texture when the water is removed. Also, dried food may suffer from adverse effects on both the color and aroma. Moreover, some drying methods, such as sun exposure, could increase the likelihood of contamination since fresh produce might be in immediate contact with various microbes (Noor Mohammed et al., 2024). Similarly, minimally processed fruits have been proposed to serve as a vector for various foodborne pathogens (Melo & Quintas, 2023). Indeed, minimally processed food has augmented increasing popularity due in part to consumer preferences for consuming fresh foods (Ete et al., 2024). Thus, these food items might require extra precautionary measures to ensure their safety for customers.

**Fig. 1:**
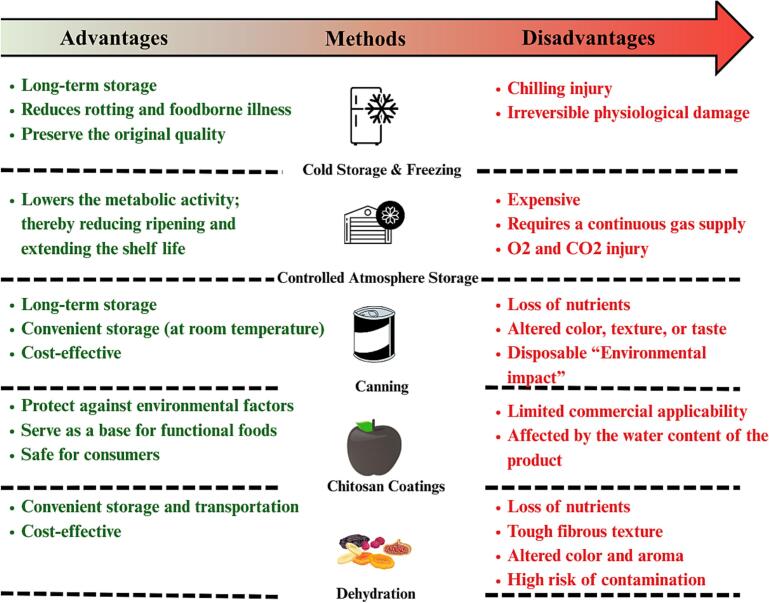
Some of the benefits and limitations of the most common preservation techniques.

Fermentation and sugaring are among the traditional methods employed to preserve fruits and vegetables (Rux et al., 2021; Swain et al., 2014; Yadav & Singh, 2014). Recently, fermentation has been linked to microbial activity and the production of biogenic amines in sufu, suggesting a potential need for further investigation into fermentation safety (Guo et al., 2025). However, these methods are still applied in certain situations and yet they may not be practical for commercial and large-scale usage due to several factors, such as restricted application, significant flavor modification, and storage availability. Although traditional preservation methods are partially successful, their ability to extend the shelf life of produce is still limited (Fig. 1). Thus, it is imperative to develop new technologies to overcome these restrictions and satisfy the increased demand for fresh produce. Novel technologies, like NPs, have the potential to provide a promising alternative solution. By combining these cutting-edge technologies with time-tested methods, preservation procedures can be enhanced to meet the increasing demands of both consumers and the food industry.

## 3 Nanotechnology applications in the food industry

In recent years, nanotechnology has been applied in the food industry and has emerged as a promising approach to enhance food quality and safety. A large body of research has demonstrated the capability of NPs to improve food processing, food packaging, nutrient delivery, and pathogen detection. In terms of food processing, NPs could be utilized to initiate new flavors or textures while reducing nutrient loss. This is achieved through the nanoencapsulation of various aromatic and flavor agents (Biswas et al., 2022). Similarly, nanoencapsulation can be used to deliver macro- and micronutrients, thereby promoting human health and alleviating chronic diseases (Altemimi et al., 2024; Pateiro et al., 2021). Considering the harsh environment of the stomach and duodenum, encapsulating bioactive compounds could maintain a stable physiological absorption rate, possibly by preventing their degradation or reducing their surface size (Awuchi et al., 2022; Grgić et al., 2020). Recently, nanoencapsulation has garnered further attention in the pharmaceutical and dietary supplements markets (Mohammad et al., 2022). This adds more economic value to nanotechnology usage in the food industry.

In food packaging, NPs have proven effective in improving the physical properties of packaging materials, thereby extending the shelf life of food items (Ashfaq et al., 2022). In fact, nanotechnology enhances the physical properties of packaging materials and provides improved protection against flame and UV light (Bajpai et al., 2018). Moreover, fresh produce is susceptible to ethylene production, oxygen sensitivity, and water permeability, all of which are key contributors to the deterioration of food quality. Therefore, improved packaging utilizing nanocoating and nano biosensors, among others, would regulate products' gas compositions and moisture levels to delay spoilage-causing factors. To that end, it is worth noting that NPs application in food packaging might be limited due in part to their high toxicity, and establishing a regulation policy for nanoparticle usage is highly recommended (Mohammad et al., 2022). Nanotechnology can also be utilized to ensure food safety, where NPs play a crucial role in enhancing nanosensors with a broad spectrum of applications (Girthie John Britto et al., 2023). Thus, nanotechnology may help provide safer food items concurrently, reducing the manpower, skills, and cost required to ensure food quality, especially in developing countries (Bülbül et al., 2015). However, technical reports are still emerging to expand nanomaterial-based fluorescents and optical immunosensor applications to improve food quality control (Guruprasath et al., 2024; Kourti et al., 2023; Zhou, Gui, et al., 2022).

## 4 Diverse NPs involved in the shelf life extension of fruits and vegetables

Recently, a large body of research has emerged to investigate the potency of utilizing NPs in preserving fresh produce. Most of these reports focus on applying NPs either during pre or postharvest. Incorporating NPs into coatings or edible films seems to be the most searched mode of application. As previously mentioned, edible coatings have been used for a long time to preserve fruits and vegetables by immersing them in coating materials to protect against moisture, oxygen, or solute movement. Although classical coating is partly successful in maintaining firmness, freshness, and nutritional profile, the large surface area of coating materials limits their potential usage and protection capacity (Ravi et al., 2022). Thus, scientists have been motivated to incorporate NPs into edible coatings mainly to enhance their barrier properties and antimicrobial capacity (Wardana et al., 2024). Indeed, biopolymers with various NPs have been made applicable and have shown beneficial outcomes compared to regular coatings. For instance, chitosan NPs synthesized with guava leaf extract exhibited a better preservation effect when applied to strawberries compared to bulk chitosan (Ali et al., 2022), further demonstrating the vital role of nanonization in preserving fresh produce.

During preharvest, NPs have been used to compensate for the potential limitations associated with traditional fertilizers, which also convey other beneficial effects, such as increasing disease resistance and improving fruit quality. NPs applied during preharvest are suggested to nourish plants effectively due in part to their small size and enhanced absorption capacity (Yadav et al., 2023). For instance, foliar applications of zinc oxide nanoparticles (ZnO-NPs) have shown improved quality parameters, concomitant with enhanced nutrient uptake in tomatoes compared to zinc oxide (Ahmed et al., 2023). Currently, the precise effect of nanofertilizers on the shelf life of fruit and vegetables is a developing research area, and the conjugated utilization of nanotechnology during pre and postharvest might open a new venue for optimizing the preservation of fresh produce. Additionally, concurrent utilization of various nanoparticles with other additives could promote a synergistic effect leading to optimized preservation outcomes (Babapour et al., 2021; Thu Bui et al., 2025). Therefore, the following section will highlight the application of the most investigated NPs in extending the shelf life of fresh fruits and vegetables, focusing on their mode of application, efficacy, benefits, and limitations.

### 4.1 Zinc oxide nanoparticles

ZnO-NPs mainly serve as antibacterial agents in the food sector and have the potential to preserve various food items (Qu et al., 2025). A large plethora of research confirmed the effectiveness of ZnO-NPs coatings and nanocomposite films in extending the shelf life of diverse fruits and vegetables (Alli et al., 2023; Chitena et al., 2023; Hmmam et al., 2023; Javad et al., 2022). These projects focus on the capability of incorporating ZnO-NPs to reduce weight loss and respiration rate, maintain firmness and nutrient content, and inhibit disease development (Table 1). Although the mechanism by which ZnO-NPs exerts their beneficial effect is still emerging, numerous reports have confirmed their impact on delaying microbial growth and promoting antioxidant capacity, which might be the primary mechanisms mediating the beneficial outcomes of ZnO-NPs (Batool et al., 2022; Chitena et al., 2023; Dulta, Koşarsoy Ağçeli, Thakur, et al., 2022; Kim et al., 2022; Nxumalo et al., 2022). The antimicrobial activity of ZnO-NPs is evident toward various bacterial strains, including *Salmonella typhimurium* (Foophow et al., 2025; Vosoughian et al., 2023). In fact, ZnO-NPs-coated low-density polyethylene films revealed high antimicrobial potential against gram-negative and gram-positive bacteria. These coated films were found to possess both short-term and long-term antibacterial capabilities, making them suitable for food packaging to enhance the quality and shelf life of fresh produce (Rokbani et al., 2019).

**Table 1:** Selected studies showing the impact of zinc oxide nanoparticles (ZnO-NPs) on fruit and vegetable preservation.

Application Type	Mode of Synthesis	NP's size (nm)	Vegetables/Fruits	Beneficial Effect on Fruits and Vegetables
ZnO-NPs-Alginate-chitosan-based coating	Chemical method	15–50 (Dulta, Koşarsoy Ağçeli, Chauhan, et al., 2022)	Oranges	↓ Weight loss ↓ Microbial growth ↓ Ascorbic acid loss ↑ Antioxidant capacity (Dulta, Koşarsoy Ağçeli, Thakur, et al., 2022)
ZnO-NPs from *Nigella sativa* extract	Biological method	2–28	Tomatoes and cucumbers	↓ Weight loss ↓ Disease development ↑ Firmness (Javad et al., 2022)
*N*,*N*,*N*-trimethyl chitosan stabilized ZnO-NPs (coating)	Chemical method	37.6	Tomatoes	- Enhance color and taste ↑ Firmness ↑ β-carotene content ↑ Shelf life (Alli et al., 2023)
ZnO-starch (nanocomposites)	Hydrothermal method	35–40	Strawberries	↓ Microbial growth of fungi *B. cinerea* and gram-positive bacteria ↓ Weight loss (Chitena et al., 2023)
Alginate-based-ZnO-NPs (coating)	Solvothermal synthesis process	12–15.1	Kiett mangoes	↓ Respiration rate ↓ Total sugars and total soluble solids (TSS) ↓ Hardness loss ↓ Weight loss (Hmmam et al., 2023)
ZnO-NPs synthesized from ginger and garlic extract (coating)	Green synthesis	40.46	Strawberries	↓ Weight loss ↓ Decay rate ↑ Antioxidant and antimicrobial capacity - Maintain firmness and ascorbic acid content (Sani et al., 2025)
Glucose oxidase (GOx)/ZnO-NPs (spraying solution)	Co-precipitation method	44.0	Peaches	↓ Weight loss ↓ TSS increase ↑ Firmness - Maintain physiological appearance - Sustain DPPH free radical scavenging activity (Batool et al., 2022)
Gum arabic (GA)-ZnO-NPs (composite coating)	Green synthesis	NA	Mandarin	↓ Electrolyte leakage ↓ Rind pitting ↓ Chilling injury ↓ Weight loss ↑ Antioxidant and phytochemical capacity (Nxumalo et al., 2022)
ZnO-NPs (Foliar application)	Chemical method	25	Tomatoes	- Enhanced quality ↑ Zinc content (Ahmed et al., 2023)
ZnO-NPs solution and ZnO-NPs + chitosan using leaf extract of cheeseweed mallow plant (*Malva Parviflora*)	Simple single-step green method	18–55	Tomatoes	↓ Weight loss ↑ Shelf life ↑ Antimicrobial activity - Enhance appearance - Better results were obtained when chitosan was added to the ZnO NPs (Iqbal et al., 2021)
ZnO-NP (Foliar application)	Chemical hydrolysis method	20–60	Melon	↑ Firmness ↑ Fruit weight ↑ Soluble solids ↑ Zinc content (Rivera-Gutiérrez et al., 2021a)
ZnO-NPs and Copper oxide (CuO) NPs coatings	Sol-gel method	NA	Guava and guava juice	- Retain the pH of the fruit ↓ Weight loss ↓ Microbial growth (Patel et al., 2022)
Coatings of carboxymethyl cellulose (CMC) and ZnO-NPs with pineapple peel extracts	Green synthesis	26–55	Persimmon and tomato	↓ Respiration rate ↓ Weight loss ↑ Fruit firmness ↑ Antioxidant capacity (Saekow et al., 2019)
Hybrid ZnO/chitosan nanocomposite	Green synthesis	45	Strawberry and red plum	↓ Weight loss ↑ Antioxidant capacity - Exhibit antibacterial properties - Maintain freshness and pleasant looking (Alamdari et al., 2022)
ZnO-NP chitosan coatings, ZnO-NPs and bergamot essential oil (BO) chitosan coating	Green synthesis	Partial size of 15	Grapes	↓ Decay incidence ↓ Microbial growth - Improve polyphenol oxidase enzyme activity - Enhance firmness and taste (Kadi, 2023)

ZnO-NPs not only function against bacteria but also have antifungal properties. Recently, copper-doped ZnO-NPs have been demonstrated to reduce the activity of *F. graminearum* under light conditions (Nan et al., 2023). Likewise, ZnO-NPs synthesized from bacteria or fungi showed antifungal capacity and prolonged the shelf life of apricot fruit (Farhana et al., 2023). These results warrant further investigation into the applicability of using ZnO-NPs to preserve tropical fruits. Moreover, ZnO-NPs showed efficacy against *Fusarium equiseti* (Subba et al., 2024), a fungus that accelerates spoilage and reduces shelf life (Bozoğlu et al., 2025; Rahman et al., 2022). Together, these studies highlighted the potential use of ZnO-NPs during preharvest to extend the shelf life of fresh produce. Various methods, such as foliar application and mineralization, aid in enhancing produce quality, thereby delaying disease development and extending storage periods (Tarafder et al., 2023). Evidently, mineralization by ZnO-NPs increased zinc content in treated tomatoes, reduced microbial growth, maintained lycopene levels, but induced water loss at higher storage temperatures (Sharifan et al., 2022). Similarly, the foliar application of ZnO-NPs to tomatoes enhanced firmness and was associated with improved quality parameters (Ahmed et al., 2023). Yet, further studies are required to evaluate the direct pre and postharvest spraying of ZnO-NPs on long-term storage.

The other mechanism by which ZnO-NPs exert their effect is through promoting antioxidant capacity. Both pre and postharvest applications of ZnO-NPs have been shown to enhance the antioxidant defense. For instance, the foliar application of ZnO-NPs at two different concentrations promoted the antioxidant capacity of melons, as evidenced by increased DPPH and vitamin C content, compared to the control and commercial fertilizers (Rivera-Gutiérrez et al., 2021b). Moreover, ZnO-NPs enhanced antioxidant capacity against cadmium and drought-induced stress in seedlings of lettuce leaves and cucumber plants, respectively (Gao, Zhang, et al., 2022; Ghani et al., 2022). However, examining the long-term effects of preharvest application of ZnO-NPs on cucumber fruits and lettuce is needed. Yet, it is worth noting that the preharvest application of ZnO-NPs failed to promote the antioxidant capacity of pomegranate arils**.** However, the postharvest application of ZnO-NPs enhanced the antioxidant activity, albeit to a lesser extent, compared to zinc sulfate (Aminzade et al., 2024). Other evidence on the efficacy of postharvest application of ZnO-NPs stems from direct treatment of ZnO-NPs or incorporating them into coating materials, where distinct reports have shown their capability to boost both enzymatic and non-enzymatic antioxidant defense (Guo et al., 2024; Nxumalo et al., 2022). Together, these studies emphasized the crucial role of ZnO-NPs in promoting antioxidant capacity.

The efficacy of using ZnO-NPs to prolong the shelf life of fresh produce is evident in various fruits and vegetables. These include but are not limited to citrus fruits (Dulta, Koşarsoy Ağçeli, Thakur, et al., 2022; Nxumalo et al., 2022), tropical fruits (Hmmam et al., 2023; Patel et al., 2022), cherries (Shahedi et al., 2024), strawberries (Alamdari et al., 2022; Chitena et al., 2023; Gao et al., 2020; Sani et al., 2025), tomatoes (Alli et al., 2023), and cucumbers (Javad et al., 2022). Apparently, incorporating ZnO-NPs into edible coatings is the most investigated method for examining ZnO-NP efficacy. Indeed, Hmmam et al. showed that the kiett mango coated with ZnO-NPs based on alginate remained firmer during storage and exhibited reduced respiration rate, total soluble solids (TSS), total sugar, and weight loss (Hmmam et al., 2023). Similarly, when compared to the uncoated control, tomatoes and cucumbers coated with ZnO-NPs synthesized from *Nigella sativa* extract exhibited a longer shelf life, accompanied by reduced weight loss, delayed illness onset, and preserved freshness (Javad et al., 2022). Moreover, coating tomatoes with ZnO-NPs can enhance their color, taste, and β-carotene content (Alli et al., 2023). Additionally, incorporating ZnO-NPs into coatings synthesized by using balangu seed mucilage mixed with gelatin and dill essential oil could optimize cherries storage under refrigeration conditions (Shahedi et al., 2024). Taken together, coating with ZnO-NPs is promising for preserving fresh fruits and vegetables.

### 4.2 Chitosan nanoparticles

Similar to ZnO nanoparticles, chitosan exhibits antimicrobial properties and thus extends the shelf life of fresh produce. These antimicrobial activities are attributed to various mechanisms, including the positively charged nature of chitosan, which enables the interaction with microorganisms, leading to increased permeability and cell death. Moreover, chitosan can inhibit the growth of pathogens by depriving microorganisms of essential elements such as metal ions, minerals, and nutrients through chelation (Maluin & Hussein, 2020). Although chitosan NPs exhibit good antimicrobial effects, they still have some limitations, including potential species specificity and sensitivity to other factors such as pH and molecular weight (Chandrasekaran et al., 2020). Therefore, incorporating chitosan with other NPs, including metal or metal oxide NPs, could further enhance the antimicrobial capacity of chitosan-based nanocomposites. This is evident in various research where chitosan or its NPs were mixed with copper (Meena et al., 2020), copper oxide (Yan et al., 2025), zinc oxide (García-García et al., 2023), silver (Shankar et al., 2021), and iron oxide NPs (Sani et al., 2024), among others, to reduce microbial growth, thereby improving fruit and vegetable storage life. Also, chitosan could be integrated with other NPs to improve its mechanical characteristics, aiding in stabilized coating and better preservation methods (Li et al., 2021). Together, these studies declare the importance of synergistic effects and synchronized utilization of various additives and NPs to optimize fruit and vegetable preservation.

Unlike other nanoparticles, chitosan is derived from chitin, a natural polysaccharide, and has been used for a long time to form edible coatings and films (Kumar et al., 2020). This provides chitosan NPs with advantageous properties compared to other NPs, which may lead to increased trust among researchers, stakeholders, and consumers, further facilitating their implementation in food preservation. As indicated previously, edible coatings and films containing chitosan NPs can preserve quality and extend the storage life of various fruits and vegetables (Al Zahrani et al., 2024; Mahmoudi et al., 2022; Meena et al., 2020; Showkat et al., 2025). In most cases, chitosan NP applications are associated with reduced weight loss, respiration rate, TSS, and enhanced nutrient content, therefore maintaining produce firmness and overall quality, as illustrated in Table 2. These beneficial observations could be attributed to the isolation capacity of chitosan NPs and their capability to promote antioxidant activity (Mahmoudi et al., 2022; Nguyen et al., 2020; Wardana et al., 2024). Indeed, incorporating *citrus aurantium* essential oil into chitosan NPs significantly induced the activity of glutathione reductase (GR) and ascorbate peroxidase (APX) concomitant with a reduction in peroxidase (POD) activity of white mushroom (Karimirad et al., 2020). Similarly, plums treated with chitosan-arginine NPs showed reduced levels of hydrogen peroxide (H2O2), parallel with increased antioxidant enzymatic activity, including catalase (CAT), superoxide dismutase (SOD), and APX (Mahmoudi et al., 2022). Thus, chitosan might have multifunctional mechanisms that aid in extending the shelf life of fresh produce.

**Table 2:** Some of the effects of chitosan nanoparticles (CTS-NPs) on preserving fruit and vegetables.

Application Type	Mode of Synthesis	Np’s size (nm)	Vegetables/Fruits	Beneficial Effect on Fruits and Vegetables
Copper-CTS-NPs	Ionic gelation method	368.3	Tomatoes	↓ Microbiological decay ↓ Weight loss ↓ Respiration rate - Preserved firmness (Meena et al., 2020)
CTS-NPs mixed with coffee residue extract (pre and post-harvest application)	Chemical method	3.9	Naples tomatoes	↓ Mycelial growth of *rhizopus stolonifer* ↓ Infection severity - Preserved firmness - Enhanced safety and shelf life (Andrea et al., 2025)
CTS-NPs coating	Ionic gelation method	290.1	Orange	↓ Weight loss ↓ Deterioration ↓ TSS to acid ratio ↓ *P. digitatum* growth (fungus) - Preserved firmness (Alshallash et al., 2022)
Packaging bags of polyethylene coated with CTS-NPs	Chemical method	60–700	Cucumber Mushroom Garlic	↓ Weight loss ↓ Mold growth - Maintain TSS ↑ Antibacterial activity ↑ Shelf life (Abu Salha et al., 2023)
CTS NPs (100 ppm) coatings with a mix of wax	Biological method	20.0	Murcott	- Maintain antioxidant capacity - Maintain vitamin C content ↑ Shelf life of Murcott (Al Zahrani et al., 2024)
CTS-NPs enriched with lemon oil	Ionic gelation method	114.62	Apples	↑ Antifungal capacity ↑ Antioxidant effect ↑ Shelf life (Dhanasekaran et al., 2025)
CTS NP films	Ionic gelation method	16.4	Apricot Fruits	↓ TSS ↓ Decay percentage ↓ Weight loss ↓ Lipid peroxidation ↑ Total acidity ↑ Ascorbic acid and carotenoid levels (Algarni et al., 2022)
CTS NP coatings pretreated with 3 % calcium chloride	Chemical method	250	Strawberries	- Improved the overall quality - Retained the contents of l-ascorbic acid and anthocyanin - Preserved antioxidant capacity ↓ Weight loss (Nguyen et al., 2020)
Arginine-coated CTS NPs	Chemical method	270	Plums	↓ Weight loss ↓ Chilling injury - Retained firmness - Enhanced ascorbic acid content, phenol accumulation, flavonoids, anthocyanin, and antioxidant capacity (Mahmoudi et al., 2022)
Proline-coated CTS NP coatings	Chemical method	∼ 250	Strawberries	- Preserve quality - Maintain phenolic content, ascorbic acid, and antioxidant capacity ↓ Weight loss ↓ Malondialdehyde and hydrogen peroxide (Bahmani et al., 2022)

Chitosan NPs alone are also beneficial in preserving fruits and vegetables (Algarni et al., 2022; Esyanti et al., 2019). For instance, chitosan NPs synthesized using the ionic gelation method showed great potential in extending the storage life of fresh apricots both at room temperature and during cold storage. Subsequently, applying chitosan NPs reduced weight loss while preserving ascorbic acid and carotenoid levels. In most cases, the increased concentration of chitosan NPs was associated with better quality outcomes compared to chitosan coating and control (Algarni et al., 2022). In addition, applying chitosan to bananas reduced the expression of ripening-related genes, thereby suppressing ethylene production. These observations were concomitant with delayed banana deterioration while maintaining sensory quality (Lustriane et al., 2018). Similarly, chitosan NPs improved storage quality measures in fresh apples, as evidenced by delayed fruit softening and weight loss while enhancing apple color (Sahraei Khosh Gardesh et al., 2016). However, in sliced apples, chitosan NPs did not change color or firmness but successfully reduced microbial activity (Pilon et al., 2015). Although most studies focused on postharvest applications of chitosan NPs, a preharvest application might be of high importance and could improve storage quality.

Preharvest utilization of chitosan NPs has shown effectiveness in improving postharvest quality parameters and extending the shelf life of fruits. The spraying of chitosan NPs, synthesized by the ionotropic gelation method, to barhi dates before harvest improved quality parameters and was associated with reduced decay proportion during cold storage compared to bulk chitosan (El-Gioushy et al., 2022). Additionally, preharvest spraying of chitosan NPs protected apples against blue mold and maintained fruit firmness, compared to chitosan or the negative control (Abdel-Rahman et al., 2021). Moreover, the foliar application of chitosan NPs to tomato leaves improved tomato yield, quality, nutrient content, and antioxidant capacity (Reyes-PÉRez et al., 2025). However, it is essential to note that using crude chitosan as a control is crucial for dissecting the precise effect of nanoization, as chitosan exhibits beneficial outcomes when applied as a foliar spray (Sajid et al., 2020). Together, these studies demonstrate the efficacy of applying pre and postharvest chitosan NPs in preserving fresh produce. Thus, an initiative strategy that integrates both methods is worth further investigation. To conclude, research has shown that chitosan NPs possess various properties and modes of application that enhance the storage duration of fruits and vegetables, primarily through their isolation capacity and antimicrobial properties, as well as their ability to boost antioxidant properties.

### 4.3 Silver nanoparticles

Compared to other nanoparticles, silver NPs exert antimicrobial activity against a broader range of pathogens (Lieu et al., 2024). Indeed, silver NPs exhibit preservation capacity even when introduce at low concentrations (Becaro et al., 2016). Therefore, various applications of sliver NPs forms such as based whey emulsions and edible films could significantly prolong the shelf life duration of fresh produce by improving the antimicrobial properties of coatings and enhancing antioxidant capacity (Rasheed et al., 2023). Further, silver NPs are effective against gram-negative and positive bacteria (Abizi-Moqadam et al., 2025) but showed higher activity toward gram-negative strains, possibly due to the differences in electrical charge (Prema et al., 2017). For this purpose, a recent study has suggested the usage of biogenic silver nanoparticles (BioAgNP) as an initiative technique to decontaminate vegetables and fruits. The application of BioAgNP alone or in combination with cinnamaldehyde (CIN) exerts antimicrobial effects against *E. coli* while maintaining the physiochemical characteristics of the tested produce (Batista et al., 2023). Together, these studies highlighted the role of silver NPs on foodborne pathogens and their potential applications to enhance fruit and vegetable safety.

Recently, numerous research has explored the efficacy of silver NPs in prolonging perishable foods' shelf life (Sun et al., 2025). In fact, perishable goods may last longer when packaged with silver NP films, as shown in Table 3**.** Indeed, the shelf life of tomatoes and coriander leaves extended significantly when wrapped with silver NP coating materials at room temperature (Kumari et al., 2021). Moreover, adding silver NPs to the pectin-gelatin matrix successfully increased the shelf life of strawberries, probably by protecting fruits against microbial and oxidant activity (Li, Fang, et al., 2025). Similarly, Gemail et al. evaluated the effect of silver NPs on murcott mandarin fruits coated with silver NPs at 50 or 100 ppm, wax, or a combination of silver NPs at 100 ppm and wax in polyethylene (PPE 0.005 %) during cold storage. During four months of storage, the combination of silver NPs and wax showed the most significant effect in reducing weight loss, wasted fruits, and enzyme activity while preserving vitamin C content and pulp firmness (Gemail et al., 2023). Furthermore, Nayab et al. demonstrated the potential role of silver NPs in regulating ethylene production, thereby extending the shelf life of bananas (Nayab & Akhtar, 2023).

**Table 3:** Efficacy of silver nanoparticles (AgNPs) in preserving fresh produce.

Application Type	Mode of Synthesis	Np’s size (nm)	Vegetables/Fruits	Beneficial Effect on Fruits and Vegetables
AgNPs-based whey emulsions and edible films. AgNPs synthesized from fungal strain- SF3	Biological method	21–62	Various fruits and vegetables	↑ Antimicrobial properties of the films and coatings ↑ Shelf life ↑ Antioxidant capacity (Rasheed et al., 2023)
AgNPs using extracts from pomegranate and watermelon peels (coating)	Green synthesis	NA	Murcott mandarin	- The combination of AgNPs and wax significantly decreased weight loss, fruit waste, catalase enzyme activity, and pulp softness although maintaining vitamin C content (Gemail et al., 2023)
AgNPs incorporated into packaging films consisting of pectin and CMC	Chemical method	28–50	Green grapes	↓ Weight loss ↑ Antibacterial activity - Maintain acidity and vitamin C content (Do et al., 2024)
AgNPs added to edible composite films and coating	Commercial	NA	Cherries	- Improved color ↑ Antimicrobial activity ↑ Preservation (Bizymis et al., 2023)
AgNPs films	Green synthesis	20	Tomatoes and coriander leaves	- Observed better shelf life of tomatoes and coriander leaves (Kumari et al., 2021)
Cellulose acetate AgNPs/TiO2	Chemical method	5.9	NA	↑ Antimicrobial action toward bacteria such as *Escherichia coli* (Jatoi et al., 2019)
AgNPs/1-MCP (composite paper)	Chemical method	420	Sweet cherries	↓ Weight loss - Enhance firmness and soluble solid content and extend the overall preservation time (Lin et al., 2022)
AgNPs incorporated into strach coating	Chemical method	12.7	Strawberries	↓ Weight loss ↓ Decay ↑ Preservation - Maintain total acidity (Taha et al., 2022)
Chitosan-based ZnO-NPs and AgNPs (coating)	Green synthesis	4.46	Papaya	↓ Weight loss ↓ Changes in TSS - Preserved ascorbic acid content - Delayed ripening - Enhanced firmness (Timilsina et al., 2025)
AgNPs	Green synthesis	10	Bananas	- Enhance banana shelf life by reducing ethylene production, thereby controlling ripeness (Nayab & Akhtar, 2023)

Recent research has focused on the biogenic and green synthesis of silver NPs to overcome safety-related issues. For instance, Shakeel et al. have utilized waste from various fruits and vegetables to synthesize silver NPs. Then, the NPs were applied to fresh fruits and vegetables, packaged in boxes made from linear low-density polyethylene, and stored for approximately two weeks. As a result, the green synthesized silver NPs enhanced firmness and extended the storage duration (Shakeel et al., 2025). Moreover, silver NPs synthesized by using tea polyphenol incorporated into sodium alginate (SA) with graphene oxide improved the properties of the composite films and showed efficacy in extending the shelf life of blueberries (Yang et al., 2024). Together, these studies revealed that silver NPs can improve the shelf duration of various fruits and vegetables by exhibiting antimicrobial properties, enhancing antioxidant capacity, improving film properties, reducing ethylene production, and enabling firmer fruits and vegetables with less weight loss.

### 4.4 Silica nanoparticles

Silica is another abundant material commonly utilized in the food industry. Recently, silica NPs have emerged as a potential mineral employed to preserve various fruits (Cao et al., 2025; Chen et al., 2025; Sami et al., 2021; Wei et al., 2023; Zhang et al., 2025a; Zhao et al., 2022). Ultimately, silica could improve the physical properties of coating and packaging materials. Also, the incorporation of silica could boost the antimicrobial and antioxidant capacity of various degradable films (Zhang et al., 2023). In fact, the enrichment of bacterial cellulose coating with silica and silver NPs augmented the coating capacity and antimicrobial action and protected against oxidative damage while reducing the weight loss of strawberries during storage (Chen et al., 2025). Moreover, adding silica NPs to nanocomposite films was suggested to improve films' thermal stability and antioxidant capacity. As a result, nanocomposite films enriched with silica NPs maintained strawberries' quality and extended their shelf life (Zhang et al., 2025b). Similarly, silica NPs significantly improved the chitosan coating of kiwi fruits and were associated with enhanced aroma during ripening (Cao et al., 2025). This indicates the potential role of silica NPs in regulating ripening, especially in hard fruits. Indeed, dendritic mesoporous silica NPs supplemented with platinum catalysts exhibited great ethylene scavenging potency concomitant with sustained banana firmness (Wei et al., 2023).

**Fig. 2:**
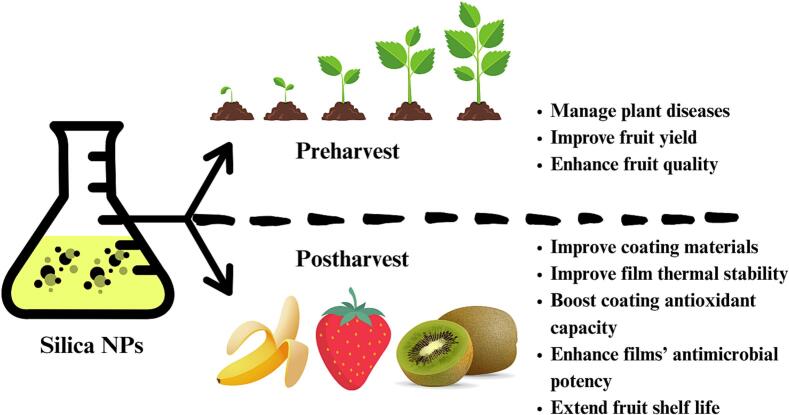
Some of the beneficial effects of silica NPs on fruit and vegetables.

Compared to fruits, only a few studies have investigated the effect of silica NPs on the postharvest preservation of vegetables. For instance, adding silica NPs to films of polylactic acid enriched with citral showed efficacy in maintaining the quality of wild mushrooms and was predicted to improve their storage (Liu, Jiang, et al., 2025). Moreover, silica NPs could play a crucial role in managing postharvest diseases. Applying silica NPs to ginger reduced decay and fungal disease concomitant with improving phenol and flavonoid content (Zhou, Liu, et al., 2022). However, silica NPs have potential applications in agriculture, thereby increasing the likelihood of improving vegetable yield, quality, and reducing disease development. This is evident in various vegetables, such as cucumber and sweet peppers, among others (Awad-Allah et al., 2021; Tajik Khademi et al., 2024). Yet, it is still imperative to determine the precise effect of silica NPs foliar treatment on postharvest preservation. Together, these studies revealed the potential role of silica NPs in preserving fruits and vegetables (Fig. 2). Moreover, compared to other NPs, silica might be advantageous due in part to its natural abundance.

### 4.5 Calcium nanoparticles

Calcium is an abundant nutrient that contributes to various essential processes critical for plants' growth and development. Calcium is a signaling molecule that regulates plant metabolism (Jing et al., 2024) and has the potency to regulate fruit senescence and ripening (Polychroniadou et al., 2024). Also, calcium is present in cell walls to provide stability and safeguard plant cells against enzymatic destruction (Jaime-Guerrero et al., 2024). Together, these provide calcium with the potential to be employed in food preservation. Indeed, calcium has acquired more popularity in preserving foods due to its natural presence and capability to enhance the structural stability of coatings and edible films. Eventually, calcium ions could improve polymer network structure by advancing crosslinking (Yin et al., 2024; Zhang, Kong, et al., 2024). Moreover, it is imperative to mention that calcium exists in various forms, such as calcium oxide, calcium carbonate, and calcium chloride (Odetayo et al., 2022), which provide calcium with distinct chemical properties and a broader application in preserving food. Thus, utilizing calcium NPs could further augment the role of calcium in preserving fresh produce.

**Fig. 3:**
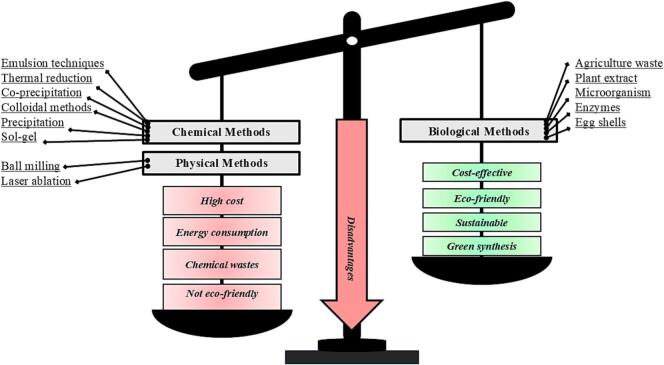
The most common methods of calcium nanoparticle synthesis.

Calcium NPs have been explored as a potential approach for conserving the quality and freshness of fruits and vegetables (Abdelkader et al., 2022; Jacuinde-Guzmán et al., 2024; Veeramani et al., 2024). Therefore, scientists have been motivated to develop various methods to synthesize calcium NPs. Apparently, the methods utilized to synthesize calcium NPs are mostly similar to other NPs, which include physical, chemical, and biological techniques (Andarini et al., 2021; Dehghani et al., 2019; Hemmami et al., 2024; Kumari et al., 2023; Ramli et al., 2019; Tang et al., 2008). Most of these methods are effective in preserving fruits and vegetables, but the biological methods using plant extract might be advantageous due to their low cost and positive environmental impact (Fig. 3). Compared to other nanoparticles, the utilization of calcium NPs might be cost-effective, environmentally friendly, and less hazardous, with positive implications for future applications (Farooq et al., 2023). To that end, successfully synthesized calcium NPs could be applied as a preharvest spray (Teixeira et al., 2022; Zhu et al., 2024) or incorporated into edible films or coatings to optimize postharvest preservation (Veeramani et al., 2022; Veeramani et al., 2024). Both approaches show efficacy in maintaining quality parameters and freshness of various fresh produce.

For a long time, calcium has been illustrated to play a critical role in preserving fruits and vegetables. For example, pre and postharvest application of calcium to red chief delicious apples preserved their texture while reducing decay onset and ethylene production. This beneficial effect was attributed to increasing calcium content, which in turn reduced physiological alteration and endogenous destruction (Conway et al., 2002; Conway & Sams, 1987). In a more recent study, preharvest calcium spraying on nanfeng tangerines increased the fruit calcium content and improved postharvest quality parameters (Chen et al., 2024). These observations encouraged scientists to further explore the potency of utilizing preharvest calcium NPs to boost fruits' calcium content, aiming to enhance quality parameters. Subsequently, preharvest spraying of calcium NPs to the leaves of red delicious apples resulted in increased calcium content, apple yield, improved quality, and delayed ripening (Ranjbar et al., 2020). Yet, further studies might be required to investigate the effect of preharvest calcium NPs spraying on the apple storage attributes. Meanwhile, preharvest spraying of calcium NPs improved table grape berries' postharvest properties, significantly reduced weight loss and decay onset, and exhibited beneficial effects during storage compared to control (Zhu et al., 2024). Together, these studies highlighted the potential impact of preharvest application on storage behavior, which could be applied alone or in combination with other preservation techniques to increase the storage period.

Compared to preharvest, numerous studies have focused mainly on employing calcium NPs during the postharvest period. After harvest, adding calcium NPs to salicylic acid inhibited enzymes responsible for cell wall destruction in cucumbers. As a result, the fruit quality was preserved concomitant with decreased weight loss and percentage of ion leakage during storage (Abdelkader et al., 2022). Similarly, in the case of mango fruit, applying a blend of calcium NPs and ascorbic acid for 24 h mitigated internal browning. This was likely due to the suppression of browning enzymes' activities and the preservation of phenolic compounds (Lo'ay & Ameer, 2019). Likewise, our recent work showed that calcium oxide NPs synthesized from *acacia arabica* leaves extended the cold storage duration of fresh-cut apples, blueberries, and blackberries. Moreover, calcium oxide NPs significantly enhanced the nutritional value of the tested fruits (Veeramani et al., 2024). Similarly, when applying calcium chloride NPs and ascorbic acid on the surface of peaches and storing them at low temperatures, calcium NPs successfully enhanced the antioxidant properties and reduced the leakage of ions in peach fruit (Lo'ay et al., 2021).

**Fig. 4:**
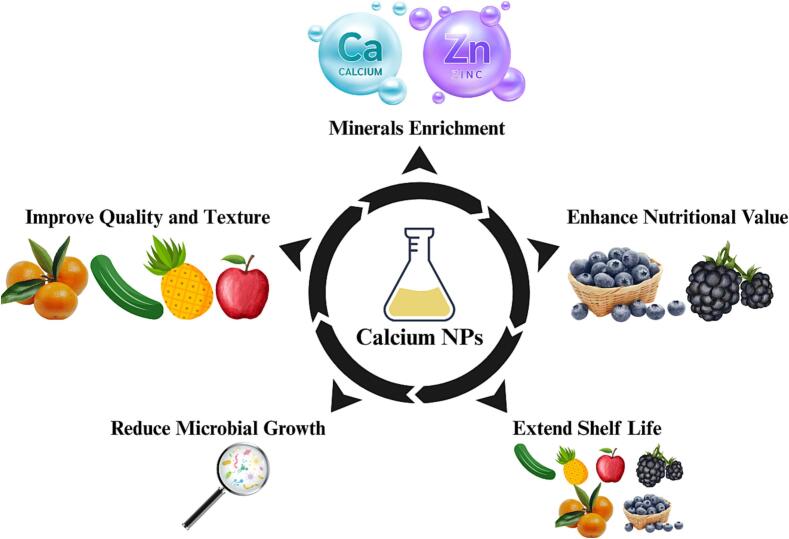
Role of calcium NPs in fruit and vegetable preservation.

As indicated previously, calcium exists in multiple forms, and choosing the appropriate form to preserve fruits and vegetables should be based on their solubility, bioavailability, potential flavor alterations (Martín-Diana et al., 2007), and antimicrobial properties (Marquis et al., 2016). Moreover, calcium oxide and calcium carbonate NPs could enhance poly methyl-methacrylate and polyethylene terephthalate coatings (Aguilera-Camacho et al., 2015; Avolio et al., 2013). Indeed, calcium oxide NPs positively affected biocompatible polymeric coatings applied on cucumbers after harvest, which improved fruit appearance and increased the amount of chlorophyll, phenolic compounds, and antioxidant capacity (Cid-López et al., 2021). Likewise, calcium carbonate NPs affected polycaprolactone-chitosan nanocomposites by stabilizing them against high temperatures and increasing their tensile strength (Abdolmohammadi et al., 2012). A recent study also showed that various concentrations of calcium carbonate NPs applied to pineapple fruit before packaging could reduce sunburn damage, improve fruit quality (including firmness and ascorbic acid content in the pulp), and make the fruit more marketable (Teixeira et al., 2022). Furthermore, most of the research on calcium NP forms has shown beneficial effects without posing harm to fruits or their plants. In conclusion, these studies revealed that various calcium NP forms could reduce microbial growth, maintain firmness, and enhance texture and nutrient contents, thereby extending the shelf life of fruits and vegetables (Fig. 4).

## 5 New trends in the preservation and packaging of fresh produce

Aside from classical and nanoparticle coating preservation methods, scientists are still motivated to establish novel techniques. Therefore, researchers are currently focusing on various approaches, including targeting ethylene scavenging and removal (Chanka et al., 2024; Lu, Mu, et al., 2025; Wei et al., 2025), developing multi-based coating and film systems (González-Cuello et al., 2024; Radan et al., 2025), and utilizing eco-friendly organic materials for safer packaging (Akhouy et al., 2025). Yet other approaches focusing on optimizing storage techniques also exist (Lu, Li, et al., 2025). In most cases, NPs have been employed in these novel strategies. For instance, Lu et al. have developed organic films containing titanium dioxide (TiO2) NPs that function as photocatalysts, thereby altering ethylene production and hindering the ripening of bananas and strawberries (Lu, Mu, et al., 2025). Based on the previous results, these films may be effective on fruits with a high ripening rate, which warrants further exploration. Moreover, a multi-system coating based on chitosan and nano zinc oxide that contained the essential oil of bergamot successfully prevented *salmonella typhimurium* growth, altered juiciness and sweetness, and, most importantly, extended red globe grapes shelf life (Kadi, 2023). Also, novel copper oxide (CuO) NPs synthesized from *ficus carica* are environment friendly and could resolve packaging for tomato preservation because they could extend the storage time of tomato fruit, maintain quality parameters, and inhibit microbes growth (Yan et al., 2025). Together, these studies highlighted the role of other NPs such as TiO2 and CuO in extending the shelf life of fresh produce.

Moreover, research aiming to advance composite films is still emerging. Recently, sustained-release composite films have been made available in the food sector and have shown potency in preserving tomatoes and mangoes, among others (Ye et al., 2025; Zhang, Pu, et al., 2024). Compared to other approaches, these films are constructed to perform several functions. First, the active components integrated into these films are released at a constant rate. Second, these films aid in preserving the nutritional value of produce and enhancing the overall quality (LaCoste et al., 2005). However, these films may require further optimization since the release rate is crucial for effective antimicrobial capacity (Cui et al., 2023; Yi et al., 2024). Nevertheless, Ye et al. successfully incorporated a nanoemulsion of cinnamon essential oil into an oxidized corn-pullulan-based matrix. The authors declared increased antimicrobial activity of the newly synthesized films concomitant with enhanced quality parameters of tomatoes during storage (Ye et al., 2025). In mangoes, incorporating ginger essential oil into sustained-release films served as active components that successfully facilitated preserving fruit quality (Zhang, Pu, et al., 2024). These studies shed light on novel composite films with potential future applications. However, it is crucial to mention that developing novel techniques should not focus solely on the efficacy of extending fresh produce but also consider their potential implementation by focusing on various aspects, such as cost, safety, and food regulations.

## 6 Conjugated utilization of nanotechnology with other preservation methods

Utilization of nanotechnology with other preservation methods might provide a new venue to optimize the preservation of fruits and vegetables. Indeed, NPs improved edible coating and showed efficacy in overcoming several limitations associated with thermal methods. For instance, chitosan arginine NPs mitigated chilling injury (CI) of plums as evidenced by reduced CI index, percentage of electrolytes leakage, and induction of malondialdehyde (MDA) content compared to controls. Yet, chitosan coating alone ameliorated CI, but the magnitude of injury reduction was higher in the NPs group (Mahmoudi et al., 2022). Moreover, treating peaches and mandarins with calcium NPs-ascorbic acid mixtures and gum arabica containing ZnO-NPs, respectively, ameliorated CI (Lo'ay et al., 2021; Nxumalo et al., 2022). Also, other NPs, including silver and calcium, showed efficacy against fruit physiological damage during cold storage (Hamouda et al., 2024; Lo'ay et al., 2021). On the other hand, NPs might be beneficial in preserving nutrient content and quality during heat sterilization of fresh produce. Utilizing ZnO-NPs with radio frequency heating (RF) preserved carrots' texture, color, and carotenoid content concomitant with enhanced antimicrobial activity compared to ZnO-NPs or RF alone (Xu et al., 2017). Moreover, dual application of chitosan NPs with laser exposure exhibited reduced microbial decay, enhanced quality parameters, and preserved vitamin C content of strawberries (Ali et al., 2022). However, it is crucial to mention that RF and laser exposure might be considered as emerging thermal techniques; thus, investigating the effect of nanotechnology on classical heating sterilization methods would be of high interest.

Aside from thermal preservation methods, nanotechnology might be beneficial once combined with other techniques such as dehydration. NPs might be associated with enhanced sensory and physiochemical parameters of dried fruits. The preharvest application of ZnO-NPs to grapes increased the overall sensory scores of sun-dried raisins but exhibited the highest moisture content compared to the negative control. Also, ZnO-NPs have been suggested as an alternative to sulfur dioxide, a well-known food additive, in raisin production (Jamali et al., 2025). Moreover, NPs could be used to advance the dehydration process, where NPs are utilized as a heat transfer fluid in closed solar dehydration systems. Indeed, the use of nanofluids containing silver NPs optimized the dehydration process and was associated with enhanced physiochemical and sensory attributes of lucuma powder compared to sun-dried samples or commercial controls (Asmat-Campos et al., 2022). Together, these results pointed to nanotechnology as a novel approach that may conserve the sensory and physiochemical properties of dried fruits. In conclusion, these studies emphasized the role of conjugated utilization of classical and modern techniques to optimize the preservation of fruits and vegetables.

## 7 Nanoparticles limitations and challenges

**Fig. 5:**
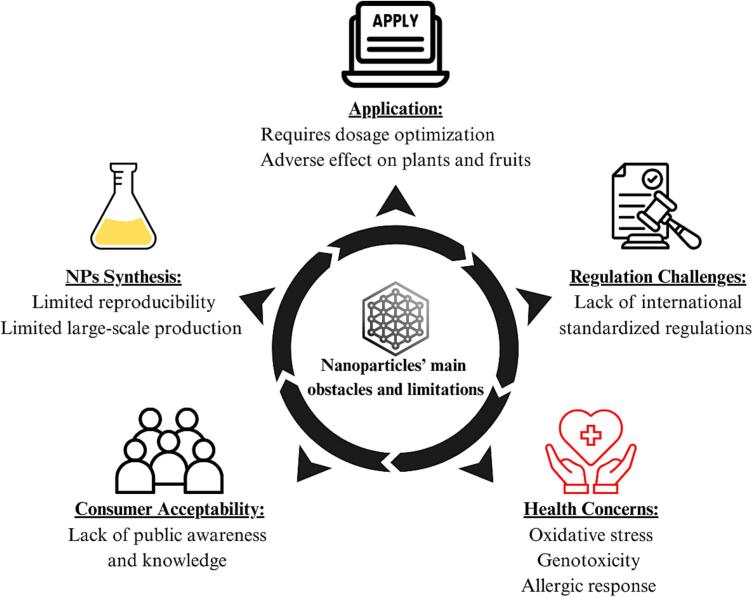
The main obstacles and challenges of nanotechnology in preserving fresh produce.

Despite the apparent beneficial outcomes of NPs in preserving fresh produce, nanotechnology is still facing serious challenges and limitations (Fig. 5). These challenges start with the synthesis process and end with consumer acceptability. In fact, NPs synthesis might be costly and energy consuming with potential solvent contamination depending on the synthesis methods (Ramanathan et al., 2021). Moreover, the reproducibility and large-scale production of NPs might be limited (Abegunde et al., 2024), highlighting the need for standardized synthesis processes and protocols. In addition, the application of NPs may have an adverse effect on fruits and vegetables, especially when applied during preharvest. In some cases, high NPs concentration is associated with reduced yield (Shahzad et al., 2024) and could hinder the growth of various plants (Hu et al., 2020; Patidar et al., 2024). Moreover, under certain conditions, NPs have been shown to impair the nutrient value of some vegetables, such as radish (Zuverza-Mena et al., 2016). Thus, optimizing the application of NPs is crucial for both plant growth and human health and could have a direct influence on consumer acceptability. Therefore, the following section will discuss nanotechnology obstacles focusing on safety, regulation, and consumer acceptability.

### 7.1 Nanoparticles and safety concerns

Although NPs have been investigated extensively on fruits and vegetables, their impact on human health is still a major concern. Certain nanoparticles, such as ZnO, are broadly searched due to their high biocompatibility and potential safety for the general public's health (Anugrah et al., 2020). Yet, consuming high amounts of ZnO could be harmful (Youn & Choi, 2022); therefore, consistent evaluation is required to ensure their safety for human consumption. Also, the safety of other metal-based nanoparticles, such as silver, is highly controversial (Kumar et al., 2025). Ultimately, extended exposure to NPs may lead to their accumulation in plants and subsequent transfer to humans, which may pose potential health risks (Sharma et al., 2022). Due to their small size, NPs are suggested to travel easily through organisms, making them accessible for cell entrance to induce oxidative stress and genotoxicity (Egbuna et al., 2021). Once oxidative stress is initiated, other cascades of biological pathways, such as inflammation and subsequently cell death, are also induced. Moreover, NPs might have a detrimental impact on vital organs such as the kidneys and lungs (Kumar et al., 2025).

Another issue associated with NPs is the possibility of triggering the immune system and subsequently inducing allergic reactions. In addition, NPs are speculated to have an adverse effect on gastrointestinal function, which may in turn promote chronic diseases associated with the immune system (Issa et al., 2022). Currently, there is a lack of knowledge regarding the transfer of NPs to fresh produce and their long-term effect on human health. Therefore, future research should not focus on the efficacy of NPs alone but must examine their safety by using *in vivo* models. Reports addressing these issues using mouse models have started to emerge (Li, Guo, et al., 2025). These reports will further advance our understanding regarding the influence of NPs on various health aspects such as appetite, overall toxicity, and NPs metabolism. Together, these concerns may have a direct impact on consumers' acceptability and could hinder the advancements of nanotechnology.

### 7.2 Insight into consumers acceptability and NPs regulation

Although many studies have provided evidence on the efficacy of NPs in preserving fruits and vegetables, consumer acceptance and food regulations may still pose a substantial challenge to advance nanotechnology in the food sector. In terms of food acceptability, consumers typically choose their food based on several factors, including cost, taste, quality, and safety (Fenyves & Kiss, 2021). Considering these factors, certain NPs might be cost-effective and could significantly enhance flavor and quality (Awlqadr et al., 2025; Liu et al., 2023). Yet, concerns regarding the safety of NPs may hinder consumers' acceptability. The hesitation in consumers' choices might be due to the limited information regarding the toxicity and safety of NPs (Gupta et al., 2024). Indeed, the lack of knowledge is a significant factor influencing consumers' acceptability, especially when introducing new food items (Laureati et al., 2024). Improving knowledge might be associated with increased confidence in nanotechnology, concomitant with reduced neophobia levels (Gómez-Llorente et al., 2022). Therefore, raising public awareness of NPs may be necessary to promote public confidence in novel preservation techniques.

Currently, there is a lack of research that measures public perceptions of nanotechnology usage, particularly in the preservation of fruits and vegetables. Only a few reports have addressed this research gap, revealing both positive and negative attitudes toward nanotechnology. For instance, the liking rate among young participants who were informed that tomatoes were preserved in packaging containing NPs was high. Further analysis using a survey revealed that most participants were willing to purchase food containing NPs for various purposes, including health benefits (Kuang et al., 2020). In contrast, a larger study conducted in Ireland showed lower consumer acceptance levels toward nanotechnology compared to conventional methods. However, health benefits and costs are suggested as a partial solution to address their concerns (Henchion et al., 2019). This discrepancy in results might be due to differences in sample age, NPs' related knowledge, or cultural background. Therefore, large-scale international studies are necessary to comprehend consumer perceptions and safety concerns.

Food regulation is another factor that could potentially influence consumers' acceptability. Reports necessitating the establishment of definitive legislation on the use of nanotechnology have started to emerge (Vega-Baudrit et al., 2024). Indeed, initial regulations have been adopted by distinct agencies (Nielsen et al., 2023; Rothen-Rutishauser et al., 2021), and various NPs have been approved for use (Adeyemi & Fawole, 2023; Siddiqui & Alrumman, 2021). These regulations focus on multiple aspects, such as transparency, whereby the disclosure of NPs content on food labels is mandatory in Europe (Rothen-Rutishauser et al., 2021). However, fresh produce is mostly sold as raw materials, which further requires additional regulation throughout the supply chain. To date, only a few NPs have been approved for food coating (Odetayo et al., 2022), indicating that the utilization and regulation of nanotechnology for fresh produce are still in their early stages. Additionally, there is a lack of studies that assess the outcomes of implementing nanotechnology in extending the shelf life of fresh produce. Addressing this research gap is crucial for evaluating the risks and benefits associated with nanotechnology. Also, it will aid in establishing better risk assessment and regulatory methods. Therefore, there is a high need to develop international standardized regulations for nanotechnology.

## 8 Conclusion

In conclusion, nanotechnology revealed significant efficacy in prolonging the shelf life of fresh produce while maintaining fruit and vegetable quality and sensory attributes. Both pre and postharvest applications of NPs using various methods showed a positive impact and successfully preserved fruits and vegetables. Additionally, integrating classical preservation methods with nanotechnology could provide a new venue for optimizing the shelf life of fresh produce. Recently, green synthetic NPs have garnered increased attention over metal-based NPs due to their stability and eco-friendly nature. Yet, dosage optimization may play a crucial role in avoiding any adverse effects of NPs applications. Therefore, implementing nanotechnology to preserve fruits and vegetables is still in its early phase. Concerns regarding the safety and regulation of NPs are a major dilemma and a developing research subject. However, to move forward, definitive regulatory measures, including globalized risk assessment models, may be necessary to evaluate the impact of NPs on human health and gain consumer trust. In this context, biological studies may be crucial in helping policymakers establish regulations and guidelines for the use of NPs. Thus, an integrated effort between researchers, consumers, stakeholders, and policymakers is essential to make nanotechnology feasible.

## CRediT authorship contribution statement

**Mohammed I. Alquraishi:** Writing – original draft. **Nora A. Alfadda:** Writing – review & editing. **Wajude A. Alabdullatif:** Writing – review & editing. **Chinnadurai Veeramani:** Writing – review & editing. **Ahmed S. El Newehy:** Writing – review & editing. **Khalid S. Al-Numair:** Writing – review & editing. **Amal A. Aloud:** Writing – review & editing. **Mohammed A. Alsaif:** Writing – review & editing.

## Declaration of generative AI and AI-assisted technologies in the writing process

During the preparation of this work the authors used Grammarly (www.grammarly.com) in order to check for spelling and grammatical errors, and to improve the manuscript without modifying the original content. After using this tool, the authors reviewed and edited the content as needed and take full responsibility for the content of the published article.

## Declaration of competing interest

The authors declare that they have no known competing financial interests or personal relationships that could have appeared to influence the work reported in this paper.

## Data Availability

No data was used for the research described in the article.
